# mRNA-Based Combination Therapy for Inflammation-Driven Osteoarthritis Induced by Monosodium Iodoacetate

**DOI:** 10.3390/pharmaceutics17101254

**Published:** 2025-09-24

**Authors:** Yuki Terai, Erica Yada, Hideyuki Nakanishi, Keiji Itaka

**Affiliations:** 1 Clinical Biotechnology Team, Center for Infectious Disease Education and Research (CiDER), The University of Osaka, Suita 565-0871, Japan; terabif@tmd.ac.jp (Y.T.); h-nakanishi@cider.osaka-u.ac.jp (H.N.); 2 Laboratory of Biomaterials and Bioengineering, Institute of Integrated Research, Institute of Science Tokyo, Tokyo 101-0062, Japan; erika.bif@tmd.ac.jp; 3 Innovation Center of Nanomedicine (iCONM), Kawasaki Institute of Industrial Promotion, Kawasaki 210-0821, Japan; 4 Nucleotide and Peptide Drug Discovery Center, Institute of Integrated Research, Institute of Science Tokyo, Tokyo 113-8501, Japan

**Keywords:** osteoarthritis, mRNA therapeutics, interleukin-1 receptor antagonist (IL-1Ra), runt-related transcription factor 1 (RUNX1), polyplex nanomicelle, intra-articular delivery, chondroprotection

## Abstract

**Background/Objectives:** Osteoarthritis (OA) is a progressive joint disease characterized by inflammation, cartilage degradation, and subchondral bone changes, for which effective disease-modifying therapies are lacking. Messenger RNA (mRNA)-based therapeutics offer a versatile approach to modulate joint pathology, but their application to OA remains limited. **Methods:** We evaluated intra-articular delivery of therapeutic mRNAs using polyplex nanomicelles, a non-inflammatory and minimally invasive carrier system, in a rat model of inflammation-driven OA induced by monosodium iodoacetate (MIA). **Results:** IL-1 receptor antagonist (IL-1Ra) mRNA reduced synovial inflammation and alleviated pain and swelling. RUNX1 mRNA, a transcription factor critical for chondrogenesis, supported chondrocyte viability, type II collagen expression, and cartilage structure. Under conditions of pronounced inflammation, however, the protective effects of RUNX1 mRNA alone were modest. Notably, combined administration of IL-1Ra and RUNX1 mRNAs produced synergistic therapeutic benefits, with enhanced chondroprotection and preservation of subchondral bone integrity. **Conclusions:** These findings suggest that while RUNX1 is essential for maintaining cartilage homeostasis, effective control of joint inflammation is required for its therapeutic activity. Dual mRNA therapy delivered by polyplex nanomicelles therefore represents a promising strategy to address the multifactorial pathology of OA.

## 1. Introduction

Osteoarthritis (OA) is a leading cause of mobility impairment among the elderly [1,2,3], affecting an estimated 350 million people worldwide [4]. While the disease is primarily characterized by progressive cartilage degeneration driven by cumulative mechanical stress, there are currently no approved disease-modifying osteoarthritis drugs (DMOADs) capable of halting or slowing the structural deterioration of joints [5,6,7,8,9].

Messenger RNA (mRNA) therapeutics have emerged as a promising modality capable of acting directly on chondrocytes within the cartilage matrix to enhance chondrogenic function [10,11,12]. We previously demonstrated that intra-articular administration of mRNA encoding the chondrogenic transcription factor Runt-related transcription factor 1 (RUNX1) effectively prevented cartilage degeneration in a surgically induced knee OA model involving resection of the medial meniscus and medial collateral ligament to simulate joint instability [13]. Following mRNA delivery, chondrocytes incorporated the transcript and exhibited enhanced chondrogenic activity, resulting in suppression of progressive cartilage degeneration. Restoration of cartilage structure at the molecular level was further confirmed using Raman spectroscopy [14].

In addition to joint instability, inflammation represents another major contributing factor in the pathogenesis of OA [15,16,17,18,19]. To address this issue, we also explored the therapeutic potential of mRNA encoding interleukin-1 receptor antagonist (IL-1Ra), a protein with well-established anti-inflammatory properties [20,21]. In a mono-iodoacetate (MIA)-induced temporomandibular joint (TMJ) arthritis model, we demonstrated that a single intra-articular injection of IL-1Ra mRNA produced sustained analgesic effects lasting for up to one month [22].

In this study, we investigated the intra-articular co-administration of IL-1Ra and RUNX1 mRNAs using polyplex nanomicelles, a safe and minimally invasive mRNA delivery platform. We hypothesized that IL-1Ra would mitigate inflammation and protect chondrocyte viability, thereby enabling RUNX1 to fully manifest its chondroprotective function, resulting in a synergistic therapeutic effect against OA progression.

The polyplex nanomicelle is our original polymer-based carrier system [23,24]. Lipid nanoparticles (LNPs) are commonly used for mRNA delivery [25,26,27] and have played a key role in mRNA vaccines, where their ability to stimulate the immune system helps improve vaccine effects [28,29,30]. However, when LNPs containing mRNA are injected into joints, we have found that they cause strong inflammatory reactions, including joint swelling and pain, immediately after injection (unpublished data). These reactions make it difficult to apply LNPs safely for OA treatment. In contrast, polyplex nanomicelles have a surface covered with a dense layer of polyethylene glycol (PEG), which helps prevent inflammation at the injection site [31]. Although immunotoxicity of PEG remains a matter of debate, it has already been widely used in pharmaceutical products, and allergic reactions have been reported to be extremely rare [32,33]. We have previously demonstrated the utility of nanomicelles for delivering mRNA not only to joints but also to other sensitive tissues, such as intervertebral disk and the central nervous system, without triggering inflammatory responses [24,34]. These applications have resulted in successful therapeutic outcomes in various disease and injury models. It is important to note that in these previous studies, we used unmodified, wild-type mRNAs rather than chemically modified forms such as pseudouridine-containing mRNA [35,36]. Despite this, we observed no significant immune responses related to mRNA immunogenicity and confirmed efficient protein expression and favorable therapeutic effects. This fact further demonstrates the capacity of the nanomicelles to avoid inflammation.

In this study, we explored the potential of administering these two therapeutic factors, RUNX1 and IL-1Ra, for the treatment of inflammation-driven knee OA. Compared to administration of either factor alone, the combination of the two factors demonstrated synergistic therapeutic effects, including pain relief and prevention of cartilage degradation. This study provides insight that highlight the potential of mRNA therapeutics for application across a variety of disease states.

## 2. Materials and Methods

### 2.1. Preparation of mRNA

Untagged open reading frames (ORFs) of human RUNX1 and human IL-1Ra were purchased from Thermo Fisher Scientific (Waltham, MA, USA). According to previous studies [13,22], mRNAs were synthesized using in vitro transcription (IVT). Briefly, each coding region was inserted into a pSP73 vector (Promega, Madison, WI, USA) containing a 120 bp poly A/T sequence downstream of the coding sequence, and transcription was driven by a T7 promoter. IVT was performed using the mMESSAGE mMACHINE™ T7 Transcription Kit (Thermo Fisher Scientific, Waltham, MA, USA) with unmodified ribonucleoside triphosphates (NTPs). Synthesized mRNAs were purified using the RNeasy Mini Kit (Qiagen, Hilden, Germany). The quantity and quality of mRNAs were assessed using a Nanodrop One spectrophotometer (Thermo Fisher Scientific, Waltham, MA, USA) and an Agilent 2100 Bioanalyzer (Agilent Technologies, Santa Clara, CA, USA). The unmodified firefly luciferase (FLuc) mRNA was purchased from TriLink (San Diego, CA, USA).

### 2.2. Synthesis of Block Copolymer

A block copolymer, polyethylene glycol- Poly(N-{N′-[N″-(2-aminoethyl)-2-aminoethyl]-2-aminoethyl}aspartamide) (PEG-PAsp(TET)) was synthesized as previously reported [37]. Briefly, β-Benzyl-L-aspartate *N*-carboxyanhydride (BLA-NCA: Chuo Kasei Co. Ltd., Osaka, Japan) was polymerized from the terminal primary amino group of α-methoxy-ω-amino poly(ethylene glycol) (PEG-NH2: Nippon Oil and Fats, Japan) (Mw 12 k) to obtain PEG-block-poly(β-benzyl-L-aspartate) (PEG-b-PBLA) by the ring-opening polymerization. Then, triethylenetetramine (TET: Wako Pure Chemical Industries, Ltd., Osaka, Japan) was introduced into the side chain of PBLA by aminolysis reaction to obtain PEG-PAsp (TET). The polymerization degree of TET was calculated as 63 using ^1^H NMR measurement (JEOL EX300 spectrometer, JEOL, Tokyo, Japan).

### 2.3. Preparation of Polyplex Nanomicelles Loading mRNAs

For preparing mRNA-loaded polyplex nanomicelles, mRNAs and PEG-PAsp(TET) were separately dissolved in 10 mM HEPES buffer (pH 7.3), then mixed them by adjusting the N/P ratio (the residual molar ratio of the polycations in amino groups to the mRNA phosphate groups) to be 3. The final mRNA concentration was adjusted to 200 μg/mL, regardless of the mRNA types. The particle size and polydispersity index (PdI) of the nanomicelles were confirmed by dynamic light scattering measurement using a Zetasizer Nano ZS (Malvern Instruments Ltd., Worcestershire, UK) (Figure S1).

### 2.4. Rat Knee OA Model

All animal experiments were approved by the Animal Care and Use Committee of Tokyo Medical and Dental University (approval no. A2022-028C5). Eight-week-old female Sprague-Dawley rats (n = 80, 180–200 g; Sankyo Labo, Japan) were housed under a 12 h light/dark cycle with free access to food and water. To induce inflammation-induced OA, monosodium iodoacetate (MIA; Sigma-Aldrich, St. Louis, MO, USA) solution was prepared at either 0.25 mg/50 μL or 1.0 mg/50 μL in saline and injected into the right knee joint under 3% isoflurane anesthesia, by inserting a 30 G needle vertically into the center of the cavity.

### 2.5. mRNA Administration into the Knee Joint

For mRNA administration into the knee joint, 50 μL polyplex nanomicelles containing 10 μg mRNA were injected into the right knee through the patellar ligament. To evaluate the protein production in the knee joint, FLuc mRNA was used, followed by measurement of luciferase protein production 24 h after the injection. Immediately after injecting 60 μL D-luciferin (50 mg/mL: Wako, Osaka, Japan), the bioluminescence was measured using the IVIS^®^ Lumina XRMS III (PerkinElmer Inc., Waltham, MA, USA) with an exposure time of 2 min. To evaluate therapeutic effects of the mRNAs on knee joints with OA, IL-1Ra mRNA and/or RUNX1 mRNA were used in the knee joints of rat OA models. For coadministration of the two mRNAs, 5 μg of each mRNA was premixed, then mixed with block copolymer solution.

### 2.6. Assessment of Knee Joint Pain and Swelling

The knee joint pain was evaluated by static weight bearing test (Incapacitance Tester, Bio Research Center, Tokyo, Japan). The load on each hindlimb was measured over a 10 s period. The pain on the MIA-injected side (right) was calculated using the following Formula (1). Each rat was tested three times per day, and the average was recorded.

(1) $$\;\;\;\;\;\;\;\%\;of\;weight\;on\;ipslateral\;limb=\frac{Right\;[g]}{Right\;\left[g\right]+Left\;[g]}$$

Knee joint swelling was evaluated by measuring anteroposterior and side-to-side diameters of the joint at the mid-level of the patellar ligament using a digital caliper. The average value of the two diameters was used to define the joint swelling.

### 2.7. Histologic and Immunohistochemical Analyses

After sacrificing the rats under deep anesthesia, the knee joint tissues were collected and quickly embedded in Super Cryoembedding Medium (Section Lab Co., Tokyo, Japan) using n-hexane. 4 µm frontal serial slices were sectioned using Kawamoto’s film method [38] and served for hematoxylin and eosin (HE) and Safranin O (SO) staining. All microscopic observations were performed using a fluorescence microscope (BZ9000, Keyence Co., Itasca, IL, USA).

Cartilage integrity was evaluated by four blinded observers independently using a scoring system provided by Osteoarthritis Research Society International (OARSI) [39]. For cell count in the cartilage, the tibial cartilage margin was manually traced, and the cartilage area was calculated using ImageJ software (version 1.53, National Institutes of Health, Bethesda, MD, USA). Within this region, the number of cells was counted from DAPI-stained images, and the results were expressed as the number of cells per unit area.

For immunohistochemical detection of FLuc, anti-FLuc primary antibody (1:50, C-12, Santa Cruz, CA, USA) was used together with Alexa Fluor 488-conjugated secondary antibody (1:500, Invitrogen, Waltham, MA, USA). For Col2 and Sox9, mouse monoclonal anti-COL2 antibody (1:100, II-4C11, Cosmo Bio, Tokyo, Japan) and rabbit polyclonal anti-SOX9 antibody (1:100, AB5535, Abcam, Cambridge, UK) were used, respectively. HRP-conjugated goat anti-mouse IgG (1:200, ab97040, Abcam, Cambridge, UK) and HRP-conjugated goat anti-rabbit IgG (1:200, ab97080, Abcam, Cambridge, UK) were applied as secondary antibodies according to standard protocols provided by the manufacturer. DAB substrate development was performed using DAB substrate kit (425011, Nichirei Biosciences, Tokyo, Japan), followed by counterstaining with Mayer’s hematoxylin and mounting with Super Cryomounting Medium Type R3 (Section Lab, Yokohama, Japan).

For relative quantification of type II collagen, 10× images of the sections stained with the above-mentioned anti-Col2 antibody were analyzed. Overall cartilage staining intensity was measured in ImageJ on an 8-bit grayscale scale (0–255). To obtain values that increase in proportion to the amount of Col2, each grayscale value was inverted (255–measured value) [40], and these inverted values were used for graphical presentation.

For the evaluation of SOX9-positive cells, tissue sections stained with an anti-SOX9 antibody were analyzed using 40× magnification images. For each animal, SOX9-positive cells were counted at two regions within the tibial cartilage, and the average of these two counts was used as the representative value for that individual.

### 2.8. μ-CT Imaging and Subchondral Bone Analysis

For analyzing the morphology of tibial subchondral bone, the knee joint was scanned using a Micro-CT Lab GX90 system (Rigaku Co., Tokyo, Japan) at indicated time points during the procedure. Under 3% isoflurane anesthesia, the rats were scanned at conditions of 90 kV, 160 μA, with a field of view (FOV) of 30 mm for 24 s. The acquired images were analyzed using 3D-BON-FCS software (RATOC System Engineering Co., Tokyo, Japan) to calculate bone volume (BV), tissue volume (TV), and bone volume fraction (BV/TV).

### 2.9. Statistical Analysis

All data are presented as mean ± standard deviation (SD). Statistical analyses were performed using EZR version 1.60 (Jichi Medical University, Tochigi, Japan), which is a graphical user interface for R (The R Foundation for Statistical Computing, Vienna, Austria) [41]. Group comparisons were conducted using one-way analysis of variance (ANOVA), followed by Tukey’s multiple comparison test. A *p*-value of <0.05 was considered statistically significant.

## 3. Results

### 3.1. Rat Knee OA Model by Injecting MIA

**Figure 1:**
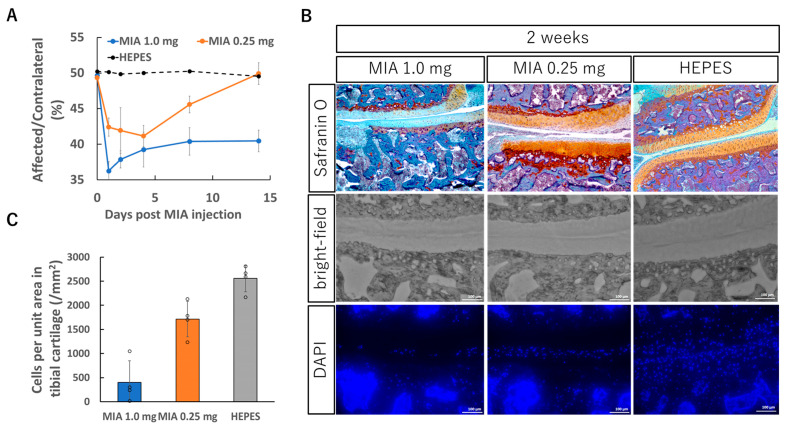
Knee OA model induced by intra-articular injection of monosodium iodoacetate (MIA). (**A**) Evaluation of knee joint pain by static weight bearing test after the MIA injection. MIA (0.25 mg or 1.0 mg) dissolved in 50 μL saline was injected into the right knee joint. The knee joint pain was evaluated for two weeks using Incapacitance Tester (Bio Research Center, Tokyo, Japan). The data are presented as the ratio of weight bearing on the affected limb relative to that on the contralateral (unaffected) limb (see Section 2). (**B**) Representative histological sections of the right knee joint 2 weeks after the MIA injection, stained with Safranin O or DAPI for nuclear staining. (**C**) Cell count in the tibial cartilage area. The tibial cartilage margin was manually traced, and the cartilage area was calculated using ImageJ software (version 1.53, National Institutes of Health, Bethesda, MD, USA). Within this region, the number of cells was counted from DAPI-stained images, and the results were expressed as the number of cells per unit area. (**A**,**C**) Data are presented as mean ± SD (n = 4 per group).

Two different conditions of MIA concentration (1.0 mg/50 μL or 0.25 mg/50 μL) were evaluated for inducing OA in the rat knee joint. The static weight bearing test to evaluate the knee joint pain indicated that both doses induced significant joint pain immediately after administration. However, the subsequent pain trajectories differed between the two groups (Figure 1A). In the 1.0 mg group, pain persisted through at least two weeks post-injection, whereas in the 0.25 mg group, pain peaked around day 4 and gradually subsided thereafter.

Histological evaluation also revealed marked differences between the groups. Knee joint tissues were collected two weeks after MIA injection and examined in frontal sections. In the 1.0 mg group, pronounced surface irregularities of the cartilage, reduced Safranin O staining intensity, and destruction of the subchondral bone were observed (Figure 1B). In contrast, although similar cartilage alterations were present in the 0.25 mg group, they were milder in severity, and changes in the subchondral bone were minimal.

Another notable difference between the groups was the number of viable chondrocytes within the cartilage tissue. DAPI nuclear staining was performed to quantify live cells in the tissue. In the 1.0 mg group, chondrocyte numbers were reduced to nearly one-tenth of those in normal cartilage, whereas in the 0.25 mg group, the reduction was limited to approximately 20% (Figure 1C). Given that the therapeutic efficacy of mRNA delivery in this study depends in part on the number of cells capable of taking up mRNA and expressing the encoded protein, this difference in cell viability is likely to be a critical factor influencing treatment outcomes.

### 3.2. Intra-Articular Delivery of FLuc mRNA in the Knee Joint with OA

**Figure 2:**
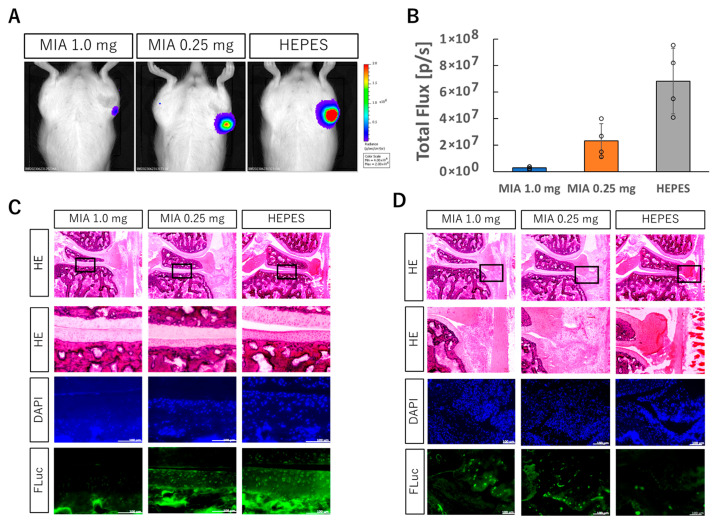
Intra-articular injection of FLuc mRNA into knee joint with OA induced by MIA injection. (**A**) Luciferase protein expression in the knee joint with OA assessed by IVIS imaging. (**B**) Quantification of bioluminescence from the IVIS images. Data are presented as mean ± SD (n = 4 per group). (**C**,**D**) Knee joint sections one day after FLuc mRNA injection: HE, DAPI, and anti-FLuc immunostaining. Enlarged views of boxed regions are shown (**C**: tibial cartilage; **D**: synovium). Scale bars, 100 μm.

To assess the feasibility of mRNA delivery into inflamed joints, firefly luciferase (FLuc) mRNA was administered using polyplex nanomicelle into the knee joints of rats two days after MIA injection (1.0 mg/50 μL or 0.25 mg/50 μL). IVIS imaging performed the following day revealed that luciferase expression in the 0.25 mg MIA group was reduced to approximately one-third of that observed in healthy cartilage, whereas expression in the 1.0 mg group was markedly diminished (Figure 2A,B). MIA is known as a highly cytotoxic compound that induces cell death and thus reduces the amount of transfectable viable tissue. Immunohistochemical analysis using an anti-luciferase antibody was then performed to assess luciferase expression specifically within the cartilage tissue. In the 1.0 mg group, luciferase-positive cells were rarely detected in the cartilage. In contrast, the 0.25 mg group showed a detectable number of luciferase-positive chondrocytes, which correlated with the higher number of viable cells observed previously (Figure 2C). Moreover, in MIA-treated joints with OA, pronounced infiltration of inflammatory cells was observed in the synovial tissue at the joint margin. These infiltrating cells exhibited high levels of luciferase expression (Figure 2D). Although a direct comparison of per-cell expression levels between healthy and OA cartilage was not performed, these results indicate that, in the 0.25 mg MIA group, polyplex nanomicelle-based delivery enables mRNA uptake and expression within chondrocytes in the osteoarthritic joint.

### 3.3. Amelioration of Joint Pain and Swelling by IL-1Ra mRNA Therapy

**Figure 3:**
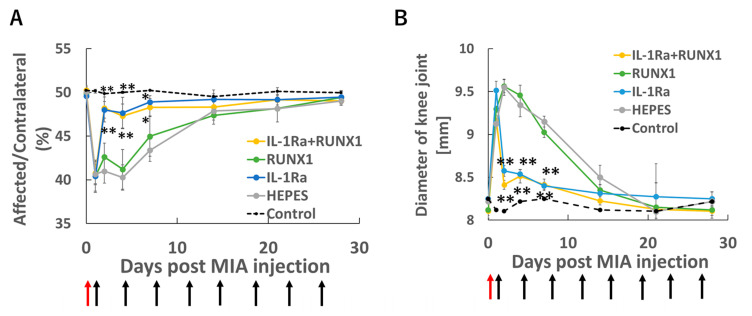
mRNA therapy in the knee joint with OA. OA was induced by intra-articular injection of MIA (0.25 mg/50 μL) on Day 0. Starting 24 h later (Day 1), therapeutic mRNAs—IL-1Ra (10 μg), RUNX1 (10 μg), or a combination of both (5 μg each)—were administered intra-articularly twice weekly. Knee pain was evaluated by static weight bearing test (**A**), and joint swelling was monitored in parallel (**B**). Red arrows indicate the time of MIA injection, and black arrows denote the timing of mRNA administrations. (**A**,**B**) Data are presented as mean ± SD (n = 8 per group). Statistical significance was determined using one-way ANOVA followed by Tukey’s multiple comparison test (* *p* < 0.05, ** *p* < 0.01 vs. HEPES).

Next, we conducted therapeutic experiments using mRNAs encoding IL-1Ra, which was expected to exert anti-inflammatory effects, and RUNX1, which was anticipated to suppress cartilage degeneration. All groups exhibited marked pain and swelling immediately after MIA injection (Figure 3A,B). In the IL-1Ra mRNA group, joint pain and swelling were significantly suppressed from the day after administration, whereas RUNX1 mRNA did not show clear symptomatic improvement. These results indicate that IL-1Ra mRNA functions effectively in alleviating inflammation-associated symptoms.

### 3.4. Effects of Runx1 mRNA and IL-1Ra mRNA to Suppress Cartilage Degeneration

**Figure 4:**
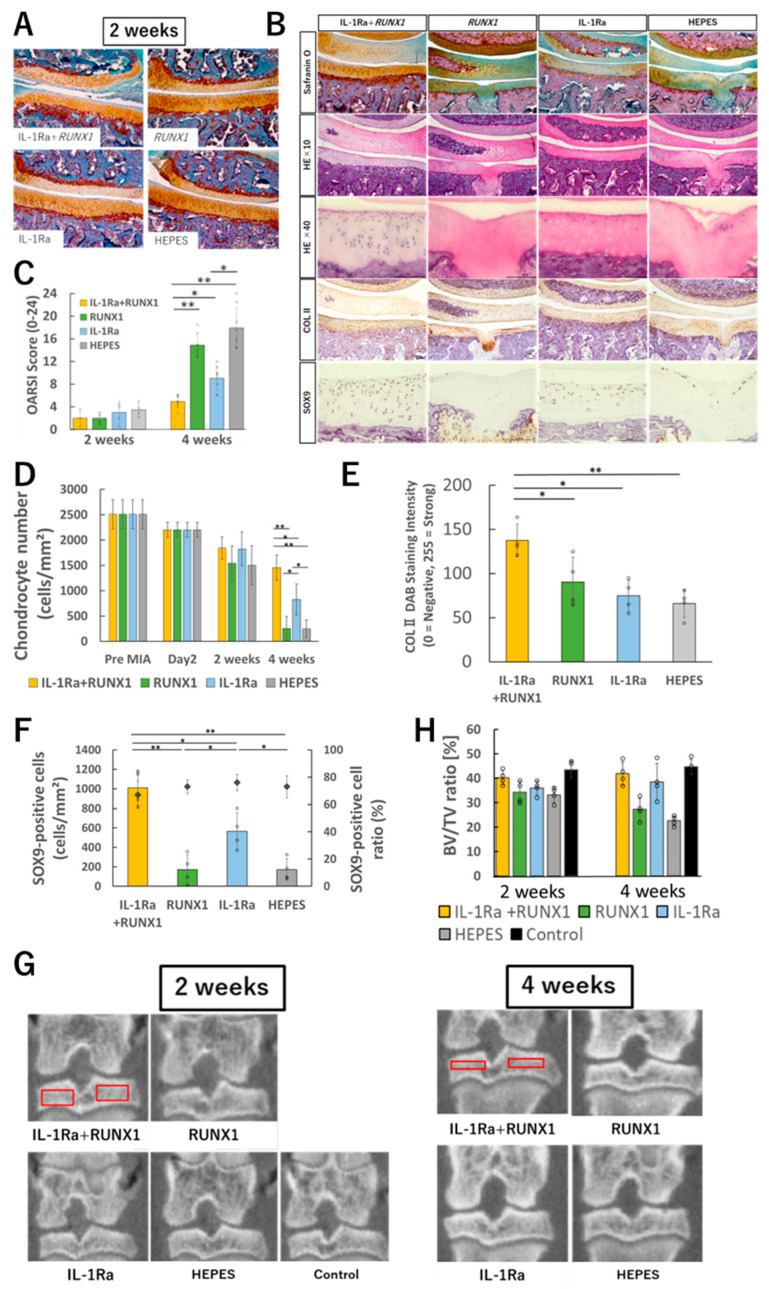
Evaluation of therapeutic effects of mRNA administration. (**A**) Representative histological images (Safranin O staining) of knee joints two weeks after OA induction by MIA (0.25 mg/50 μL) and subsequent intra-articular administration of therapeutic mRNAs (IL-1Ra, RUNX1, or both). (**B**) Histological images at four weeks after treatment initiation. From top to bottom: Safranin O staining, HE staining at low magnification, HE staining at high magnification, immunohistochemical staining with anti-type II collagen antibody, and immunohistochemical staining with anti-SOX9 antibody. (**C**) OARSI scoring based on histological evaluation (n = 8 per group). (**D**) Quantification of chondrocyte number in tibial cartilage (n = 4). (**E**) Quantification of type II collagen in tibial cartilage (n = 4). (**F**) Quantification of SOX9-positive cells in tibial cartilage (n = 4). (**G**) Representative μ-CT images of the knee joint. Red boxes indicate the regions of interest for bone volume fraction (BV/TV) measurement. (**H**) Quantitative analysis of bone volume fraction (BV/TV) (n = 4). Data are presented as mean ± SD. Statistical significance was determined using one-way ANOVA followed by Tukey’s multiple comparison test (* *p* < 0.05, ** *p* < 0.01). Scale bars for histological sections, 100 μm.

The effects of the two mRNAs on suppressing cartilage degeneration in joints with OA (induced by MIA 0.25 mg/50 μL) were evaluated histologically. Similarly as Figure 2B, at two weeks post-MIA injection, no apparent signs of cartilage or subchondral bone degeneration were observed in any group, including the untreated control (Figure 4A and Figure S2). Consistently, there were no significant differences in OARSI scores among the groups at this time point (Figure 4C).

In contrast, by four weeks post-MIA injection, the untreated group exhibited pronounced degenerative changes, including surface erosion of cartilage, reduced Safranin O staining, and subchondral bone destruction (Figure 4B and Figure S3). Similar subchondral bone alterations were also seen in the RUNX1 mRNA alone group. However, in the IL-1Ra mRNA group, these changes were notably milder, and in the combination treatment group, Safranin O staining was largely preserved with minimal degenerative changes observed. Quantitatively, the OARSI score was significantly improved in the IL-1Ra mRNA group compared to the untreated control group, and even more significantly improved in the combination group compared to the IL-1Ra mRNA group alone (Figure 4C).

Quantification of chondrocyte numbers within the cartilage revealed a gradual decrease in all groups up to two weeks after MIA injection. Thereafter, a sharp decline in cell numbers was observed in both the untreated and RUNX1 mRNA groups (Figure 4D). In contrast, the IL-1Ra mRNA group showed a significantly attenuated rate of cell loss, and in the combination treatment group, chondrocyte numbers remained largely stable after the two-week mark. Notably, the combination group exhibited a trend toward higher cell density in the deep zone of the cartilage compared to the IL-1Ra mRNA group alone. A similar pattern was observed in the quantification of type II collagen, which was better preserved in the combination group than in any other group, suggesting enhanced maintenance of the cartilage matrix (Figure 4E).

To assess the chondrogenic potential of surviving chondrocytes, immunostaining with an anti-SOX9 antibody was performed. The percentage of SOX9-positive cells among total chondrocytes remained above 70% across all groups, indicating a preserved differentiation phenotype (Figure 4F). However, due to group differences in total cell number, the absolute number of SOX9-positive cells per unit area varied markedly among groups.

To evaluate the impact of inflammatory OA on subchondral bone, bone volume analysis using μ-CT was performed. While no significant differences were observed among groups at two weeks post-MIA injection, by four weeks, the untreated and RUNX1 mRNA alone groups showed substantial reductions in bone mineral density (Figure 4G,H). In contrast, this bone loss was significantly attenuated in both the IL-1Ra mRNA and combination treatment groups.

Taken together, these findings demonstrate that, when the two were combined, IL-1Ra reduced inflammation and protected chondrocytes, thereby enabling RUNX1 to fully manifest its chondroprotective effects. This complementary interaction was reflected in significantly improved OARSI scores, higher chondrocyte density, and superior preservation of cartilage matrix in the combination group compared with either monotherapy.

### 3.5. mRNA Administration in Joints with Severe OA

**Figure 5:**
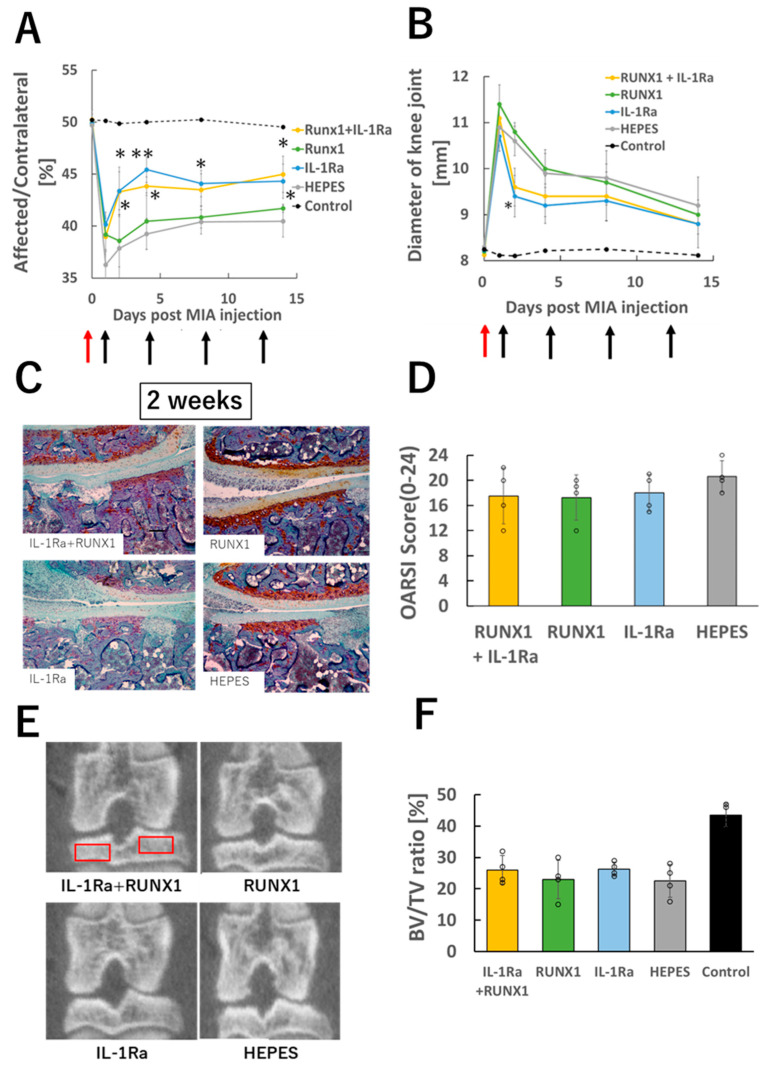
mRNA therapy in knee joints with severe OA. (**A**) Knee pain assessed by static weight bearing test two weeks after OA induction with high-dose MIA (1.0 mg/50 μL). (**B**) Evaluation of knee joint swelling. Red arrows indicate the time of MIA injection, and black arrows denote the timing of mRNA administrations. (**C**) Representative histological images of knee joints (Safranin O staining). (**D**) OARSI scoring based on histological images (n = 4 per group). (**E**) Representative μ-CT images of the knee joint. Red boxes indicate the regions of interest for bone volume fraction (BV/TV) measurement. (**F**) Quantitative analysis of bone volume fraction (BV/TV) (n = 4). Data are presented as mean ± SD. Statistical significance was determined using one-way ANOVA followed by Tukey’s multiple comparison test (* *p* < 0.05, ** *p* < 0.01 vs. HEPES).

Finally, we tested the therapeutic effects of IL-1Ra mRNA and RUNX1 mRNA in a severe OA model induced by a high dose of MIA (1.0 mg/50 μL), as described in Section 3.1. On Day 1, joint pain was even more pronounced than that observed in the lower-dose (0.25 mg/50 μL) model (Figure 5A). Although IL-1Ra mRNA administration resulted in mild pain relief, it was not sufficient to fully resolve the symptoms. Joint swelling also persisted through Day 14 (Figure 5B).

Histological analysis at two weeks post-MIA injection revealed widespread loss of Safranin O staining and severe subchondral bone destruction in all groups. Chondrocytes were almost completely absent from the cartilage tissue (Figure 5C). Consistently, OARSI scores were elevated across all groups (Figure 5D), and micro-CT analysis showed significant reductions in bone volume in every group (Figure 5E,F). These results indicate that the intense joint inflammation caused by high-dose MIA may not be amenable to treatment with mRNA therapeutics.

## 4. Discussion

Osteoarthritis (OA) is a multifactorial disease involving mechanical degradation and activation of inflammatory cytokines. Consequently, therapeutic strategies that target multiple disease pathways are essential. This study demonstrated that mRNA therapeutics are effective in knee osteoarthritis characterized by inflammation, and that the combined administration of multiple factors can effectively address the complexity of this condition.

Our results in the low-dose (0.25 mg/50 μL) MIA-induced OA model highlight the central role of RUNX1 as a disease-modifying factor in OA. RUNX1 mRNA enhanced chondrocyte survival, promoted the expression of cartilage-specific markers such as type II collagen and SOX9, and preserved cartilage architecture. However, in inflammation-driven OA, RUNX1 alone was insufficient to relieve joint pain or prevent extensive chondrocyte loss, likely due to the hostile inflammatory milieu that rapidly depletes viable target cells. IL-1Ra mRNA, by contrast, exerted rapid anti-inflammatory effects, reducing joint pain and swelling within days. Although IL-1Ra did not directly restore cartilage integrity, it played an essential enabling role by preserving the cellular environment required for RUNX1 to function. When both mRNAs were co-administered, IL-1Ra suppressed inflammation and apoptosis, while RUNX1 promoted chondrocyte differentiation and matrix synthesis. This complementary interplay resulted in superior outcomes compared to either monotherapy, underscoring the mechanistic rationale of the combination approach.

Importantly, the combination therapy significantly reduced cell death in the deep cartilage layer, which is particularly susceptible to damage in OA due to limited nutrient diffusion and mechanical stress [42,43,44]. RUNX1 is known to enhance the synthesis of extracellular matrix components such as type II collagen and to confer mechanical resilience to cartilage. Additionally, it promotes cell survival and modulates anti-apoptotic pathways, potentially contributing to the preservation of deep-zone chondrocytes [45,46].

In the high-dose (1.0 mg/50 μL) MIA model, however, IL-1Ra mRNA conferred only modest pain relief, with no apparent improvement in cartilage preservation or bone integrity compared to the low-dose group. The exacerbated inflammatory milieu likely diminished the number of viable target cells and triggered antiviral responses—such as PKR activation—that can inhibit mRNA translation and diminish therapeutic efficacy [47,48]. Nevertheless, because IL-1Ra is a secreted protein, its expression by non-chondrocytic cells may have contributed to the observed, albeit limited, anti-inflammatory effects [49]. These findings underscore the importance of disease stage and inflammatory status in determining the effectiveness of mRNA-based interventions.

Because OA is a chronic disease that progresses with aging, repeated administration of therapeutic agents is unavoidable. However, compared with recombinant protein formulations such as IL-1Ra, which have a short half-life [50], mRNA therapeutics offer design flexibility and enable sustained local protein production, which may help reduce the frequency of administration. Moreover, mRNA allows for co-delivery of multiple therapeutic agents, offering integrated control of inflammation and tissue repair. However, in the context of severe inflammation, intracellular antiviral responses can inhibit mRNA translation, and reduced target cell availability limits efficacy. Thus, optimizing treatment timing and delivery strategies is essential. Additionally, challenges related to manufacturing costs and storage stability must be addressed.

The MIA-induced OA model used in this study is well-suited for investigating osteoarthritis accompanied by acute inflammation. In particular, the low-dose model reproduces certain features of human OA, including progressive joint degeneration that continues even after an initial inflammatory insult, without the need for ongoing external triggers. This disease trajectory resembles the sustained cytokine activation observed in some human OA cases [51,52,53]. However, MIA is a highly cytotoxic compound that induces cell death by disrupting cellular metabolism [54,55,56] and thus does not accurately reflect the typically slow and progressive nature of OA. This represents one of the limitations of the present study. Furthermore, the MIA model does not fully replicate chronic OA driven by joint instability or biomechanical stress [57,58]. Therefore, future studies should evaluate the efficacy of this therapeutic strategy using alternative models—such as surgically induced or aging-associated OA models—that better represent the diverse pathophysiological mechanisms underlying human OA.

## 5. Conclusions

This study provides evidence that combining mRNAs targeting distinct OA pathophysiology can effectively slow disease progression. RUNX1 is the primary driver of structural modification in OA, whereas IL-1Ra serves as a critical enabler that unlocks the therapeutic potential of RUNX1 under inflammatory conditions. This driver–enabler paradigm provides a new conceptual framework for mRNA therapeutics in OA, highlighting the importance of tailoring combination strategies to disease stage and inflammatory status.

## Data Availability

The data presented in this study are available on request from the corresponding author.

## Supplementary Materials

The following supporting information can be downloaded at: https://www.mdpi.com/article/10.3390/pharmaceutics17101254/s1. Figure S1: Characterization of polyplex nanomicelles: particle size and polydispersity index (PDI); Figure S2: Additional Safranin O staining images of articular cartilage 2 weeks after MIA and mRNA administration; Figure S3: Additional Safranin O staining images of articular cartilage 4 weeks after MIA and mRNA administration.
