# Supplementary material for: mRNA-Based Combination Therapy for Inflammation-Driven Osteoarthritis Induced by Monosodium Iodoacetate
Source: Pharmaceutics. 2025 Sep 24;17(10):1254. doi: 10.3390/pharmaceutics17101254 (PMC12567188; doi:10.3390/pharmaceutics17101254)
Supplement: Supplementary file 1 [file pharmaceutics-17-01254-s001.zip › pharmaceutics-3874740-supplementary.pdf]

pharmaceutics

MDPI

Article

mRNA-Based Combination Therapy for Inflammation-Driven Osteoarthritis Induced by Monosodium Iodoacetate

Yuki Terai [1,2,3], Erica Yada [2,3,4], Hideyuki Nakanishi [1,2] and Keiji Itaka [1,2,3,4,*]

[1] Clinical Biotechnology Team, Center for Infectious Disease Education and Research (CiDER), The University of Osaka, Osaka 565-0871, Japan; terabif@tmd.ac.jp (Y.T.); h-nakanishi@cider.osaka-u.ac.jp (H.N.)
[2] Laboratory of Biomaterials and Bioengineering, Institute of Integrated Research, Institute of Science Tokyo, Tokyo 101-0062, Japan; erika.bif@tmd.ac.jp (E.Y.)
[3] Innovation Center of Nanomedicine (iCONM), Kawasaki Institute of Industrial Promotion, Kanagawa 210-0821, Japan
[4] Nucleotide and Peptide Drug Discovery Center, Institute of Integrated Research, Institute of Science Tokyo, Tokyo 113-8501, Japan
* Correspondence: itaka@cider.osaka-u.ac.jp; Tel.: +81-6-6879-8889

Supplementary Materials

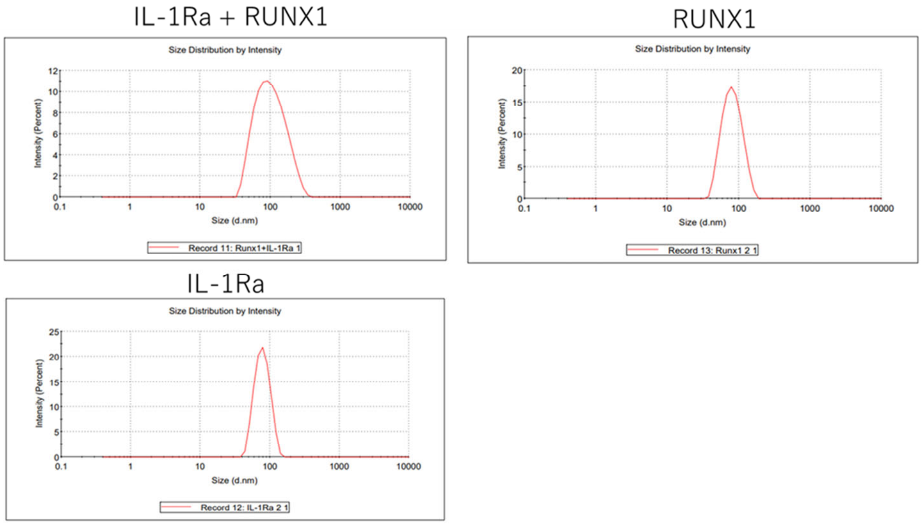

	Z-Average [nm]	PdI
IL-1Ra + RUNX1	89.50	0.191
RUNX1	77.80	0.124
IL-1Ra	74.45	0.068

Figure S1. :Characterization of polyplex nanomicelles: particle size and polydispersity index (PDI).

 https://doi.org/10.3390/pharmaceutics17101254

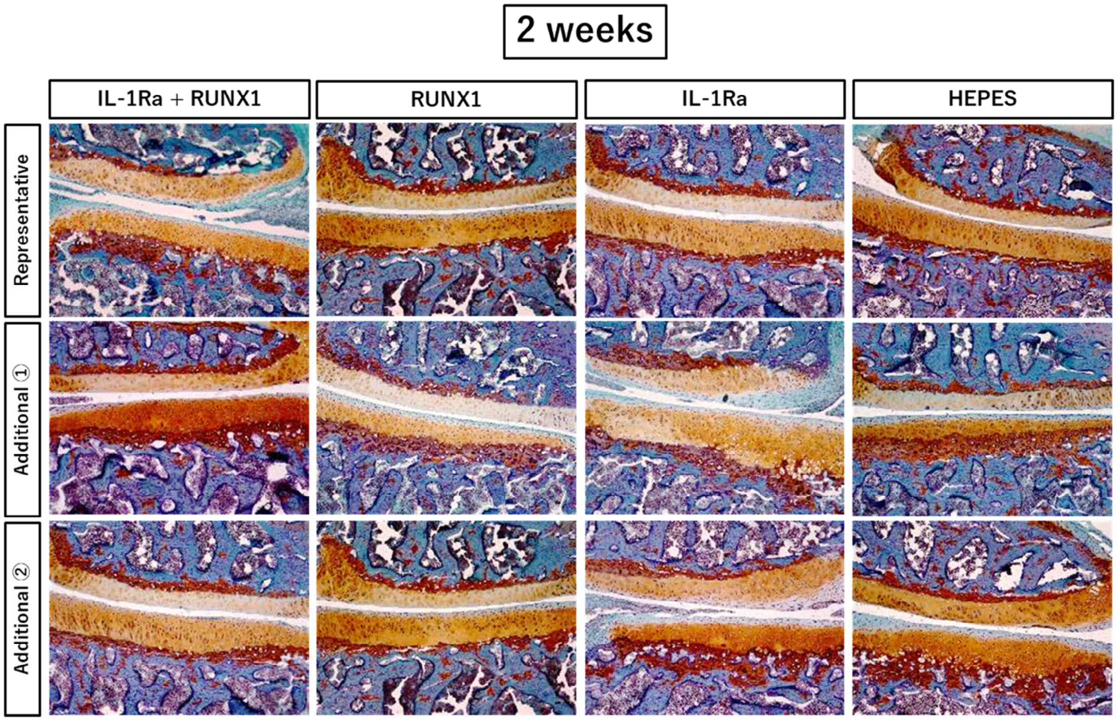

Figure S2. : Additional Safranin O staining images of articular cartilage 2 weeks after MIA and mRNA administration.

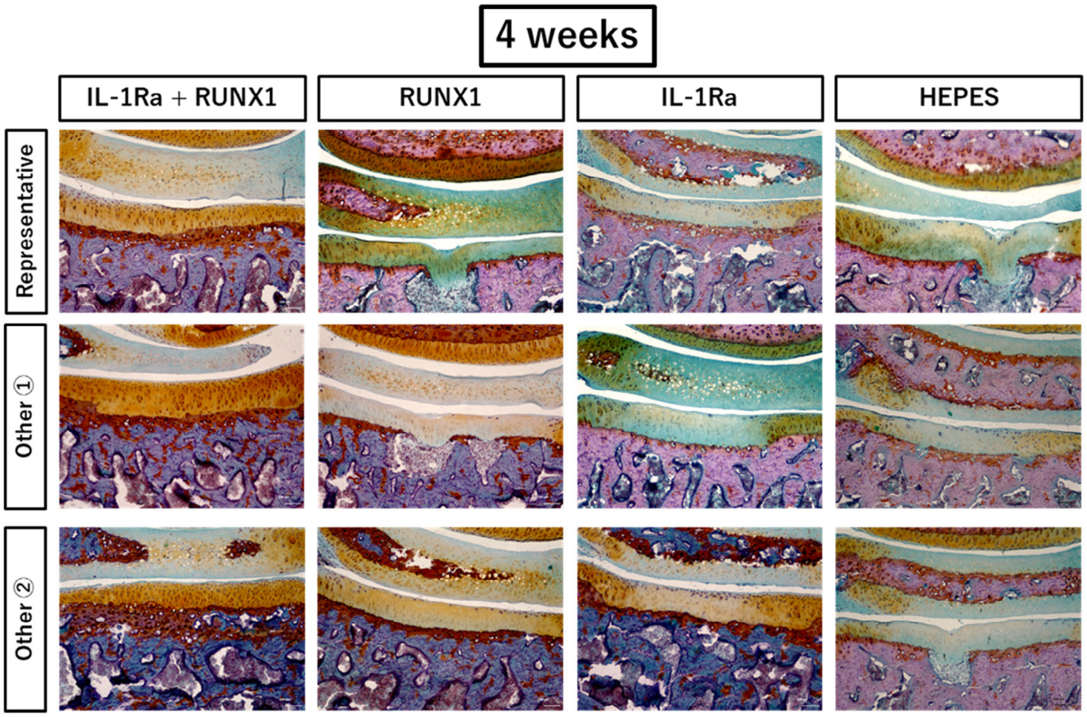

Figure S3. : Additional Safranin O staining images of articular cartilage 4 weeks after MIA and mRNA administration
